# The vestibular system modulates the contributions of head and torso to egocentric spatial judgements

**DOI:** 10.1007/s00221-021-06119-3

**Published:** 2021-06-04

**Authors:** Elisa R. Ferrè, Adrian J. T. Alsmith, Patrick Haggard, Matthew R. Longo

**Affiliations:** 1 Department of Psychology, Royal Holloway, University of London, London, UK; 2 Department of Philosophy, King’s College London, London, UK; 3 Institute of Cognitive Neuroscience, University College London, London, UK; 4 Department of Psychological Sciences, Birkbeck, University of London, London, UK

**Keywords:** Egocentric representation, Galvanic vestibular stimulation, Vestibular system, Misalignment paradigm, Multisensory integration

## Abstract

Egocentric representations allow us to describe the external world as experienced from an individual’s bodily location. We recently developed a novel method of quantifying the weight given to different body parts in egocentric judgments (the *Misalignment Paradigm*). We found that both head and torso contribute to simple alter-egocentric spatial judgments. We hypothesised that artificial stimulation of the vestibular system would provide a head-related signal, which might affect the weighting given to the head in egocentric spatial judgments. Bipolar Galvanic Vestibular Stimulation (GVS) was applied during the Misalignment Paradigm. A Sham stimulation condition was also included to control for non-specific effects. Our data show that the weight given to the head was increased during left anodal and right cathodal GVS, compared to the opposite GVS polarity (right anodal and left cathodal GVS) and Sham stimulation. That is, the polarity of GVS, which preferentially activates vestibular areas in the right cerebral hemisphere, influenced the relative weightings of head and torso in egocentric spatial judgments.

## Introduction

When describing our surroundings we may need to use expressions such as “on my left”, “on my right”, etc*.*, to facilitate the hearer’s imagination of a scene, or mark an important contrast between the spatial relations of the speaker and the hearer relative to a common environment. In doing so, we are making use of a common cognitive resource, the capacity for *egocentric spatial representation*. Egocentric representations describe the external world as experienced from an individual’s location, in a manner sensitive to how the individual’s body is disposed (Jeannerod and Biguer, 1987). The body is therefore considered the point of origin of egocentric representations (Bermúdez 1998, 2005). Critically, however, bodies are not points; they are extended three-dimensional objects with articulated joints and independently mobile parts. Changes in body posture potentially dissociate reference frames anchored to different body parts. Thus, different body parts may function as origins of the egocentric reference frame.

The contribution of individual body parts in influencing egocentric spatial judgments is highlighted by Peacocke’s (1992) Buckingham Palace thought experiment (p. 62):

“Looking straight ahead at Buckingham Palace is one experience. It is another to look at the palace with one’s face still toward it but with one’s body turned toward a point on the right. In this second case, the palace is experienced as being off to one side from the direction of straight ahead, even if the view remains exactly the same as in the first case.”

This example captures the intuition that changes of body orientation can dissociate the relative spatial relations of objects to different body parts, highlighting the problem of which body part—if any—serves as the origin of egocentric representations. Both the head and torso are strong candidates for this role. On one side, the head hosts the majority of sensory organs—the eyes, ears, and the vestibular receptors—which provide a constant flow of afferent sensory information (Sherrington 1907; Avillac et al. 2005). On the other side, the torso is probably the most stable anchor for the construction of a consistent egocentric representation (Karnath et al. 1991; Serino et al. 2015), as the “great continent” of the body (Alsmith and Longo 2014).

**Fig. 1:**
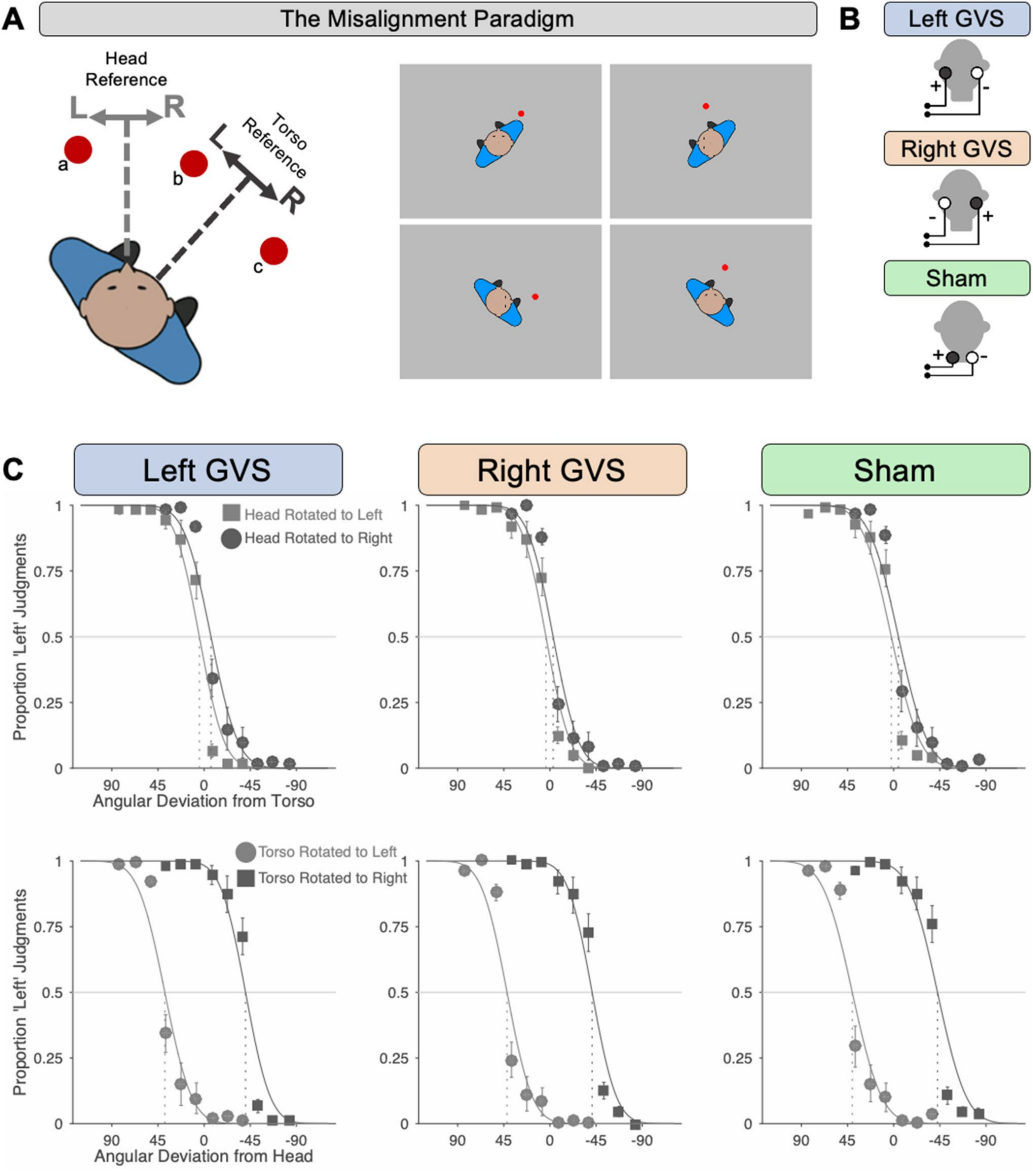
Experimental set up and results. **A** The Misalignment Paradigm (adapted from Alsmith et al. 2017). The locations of balls a and c are clearly at the left and right of the person, while the location of ball b is critical; if the torso is the origin of the egocentric reference frame, ball c is to the person’s left; if the head is the origin, it is to their right. **B** Examples of visual stimuli used in the Misalignment Paradigm. **C** GVS configuration. Both left anodal and right cathodal (Left GVS) and right anodal and left cathodal (Right GVS) configurations were used. A Sham stimulation with the electrodes placed on the left and right side of the neck was adopted to control for non-specific effects. **D** Proportion of ‘Left’ judgements as function of angular deviation from the torso and head. The weight given to the torso was overall greater than that given to the head. Critically, artificial vestibular stimulation modulated these weights; Left GVS increased the weighting given to the head compared to Right GVS or Sham stimulation Alsmith et al. (2017)

We have recently developed a *Misalignment Paradigm* which isolates the respective contribution of the head and torso to egocentric spatial judgments (Alsmith et al. 2017; Longo et al. 2020). This paradigm is essentially an experimentalization of Peacocke’s (1992) Buckingham Palace thought experiment, described above. We showed participants a bird’s eye view of an avatar whose head was turned 45° to the left or right of the torso and asked them to judge whether objects were “to the person’s left” or “to the person’s right” (see Fig. 1A). By measuring how these judgments change as a function of the position of the object relative to the head and torso, we determined the contributions of each body part to egocentric spatial judgments. Our results suggested that both head and torso contribute to egocentric spatial judgments, though with greater weight given to the torso in most participants. Interestingly, individual differences in the weighting of the two body parts were correlated across different spatial axes and stable over time (Longo et al. 2020).

It remains unclear, however, whether the contribution of the head and torso to egocentric spatial judgments is a *static* stored representation reflecting primarily semantic knowledge about body morphology, or a *dynamic*, continuously updated sensory representation, perhaps reflecting the saliency of afferent sensory signals. Here we investigated whether the weight given to the head and torso in the Misalignment Paradigm could be manipulated by changing the saliency of afferent sensory signals.

Visual, auditory, proprioceptive and vestibular signals could all contribute to egocentric spatial judgements (Jeannerod 1988; Blouin et al. 1996, 1998). However, the vestibular signals seem to be particularly relevant (Schilder 1935; Lhermitte 1952; Bonnier, 1905). As Sherrington (1907) noted, the vestibular system is a significant source of information about self-orientation, given that it “*maintains not merely a limb in flexion or extension, but a posture of the whole animal in regard to gravitation*” (p. 480). The vestibular system is a sophisticated set of sensory transducer organs that respond to motion of the head. It comprises three orthogonal semi-circular canals (anterior, posterior and horizontal) that sense rotational acceleration of the head in three-dimensional space, around the yaw, roll, and pitch axes, and two otolith organs (the utricle and saccule) that sense translational acceleration, including the orientation of the head relative to gravity. The vestibular system provides an important reference for control of the head in space (Lackner and DiZio 2005); any movement of the head generates a flow of vestibular acceleration signals which are integrated with input from other sensory modalities from vision and neck muscles. Not surprisingly, the vestibular system is highly interlinked with both visual and proprioceptive systems, with a large number of thalamic neurons responding to both vestibular, visual and proprioceptive inputs (Deecke et al. 1977; Schwarz et al. 1973). The cortical vestibular system is also strongly integrated with other sensory modalities. Neuroimaging studies have identified a widespread vestibular network in the human brain, which includes the Temporo-Parietal Junction (TPJ), posterior insula, superior temporal gyrus, Inferior Parietal Lobule (IPL), Anterior Cingulate Cortex (ACC), fronto-parietal operculum, both primary and secondary somatosensory cortices and the prefrontal cortex (Lopez et al. 2012; Zu Eulenburg et al. 2012). Functional responses suggest that the posterior parietal operculum is the core area of the human vestibular network (Eickhoff et al. 2006a, b). Critically, this area contains not only the representation of the body, but also accurate body-in-the-worlds maps (Knox et al. 2006).

Vestibular signals play a role in determine the location of environmental objects in respect to the body (Clement et al. 2009) and are of central importance in structuring individuals’ experience of the world in relation to themselves and others (Lopez et al. 2010; Deroualle and Lopez, 2014; Lenggenhager and Lopez 2015; Pavlidou et al. 2018). We hypothesised that artificial stimulation of the vestibular system would provide a head-related acceleration signal, which might increase the saliency of head-related signals, and therefore affect the weighting given to the head in performance of the Misalignment Paradigm (Alsmith et al. 2017). We have used low-intensity bipolar Galvanic Vestibular Stimulation to non-invasively stimulate the vestibular receptors (Fitzpatrick and Day, 2004). An anode and cathode were placed on the left and right mastoid, or vice versa (Fig. 1B). Perilymphatic cathodal currents depolarize the trigger site and lead to excitation, whereas anodal currents hyperpolarize it resulting in inhibition (Goldberg et al. 1984). Galvanic Vestibular Stimulation causes polarity-dependent behavioural effects which are consistent with neuroimaging evidence revealing asymmetrical cortical vestibular projections in the non-dominant hemisphere of right-handed participants (Dieterich et al. 2003). We investigated whether a Galvanic Vestibular Stimulation induced bias on spatial egocentric judgments supports a *dynamic* sensory-driven egocentric representation.

## Methods

### Participants

Fifteen right-handed individuals (eight women) between 19 and 34 years (mean age ± SD: 23.8 ± 3.4 years) participated. The sample size was decided a priori based on similar experiments (Alsmith et al. 2017; Ferrè et al. 2013). The sample size was set in advance of testing and was also used as data-collection stopping rule. All participants were right-handed (Edinburgh Handedness Inventory, Oldfield, 1971) with normal or corrected-to-normal vision. Exclusion criteria included neurological, psychiatric or vestibular conditions, epilepsy or family history of epilepsy. The experimental protocol was approved by the Department of Psychological Sciences research ethics committee at Birkbeck, University of London. The study adhered to the ethical standards of the Declaration of Helsinki. Participants gave written informed consent to participate before inclusion in the experiment.

### Galvanic vestibular stimulation

Bipolar Galvanic Vestibular Stimulation (GVS) was applied to deliver a boxcar pulse of 1 mA for 3 s using a commercial stimulator (Good Vibrations Engineering Ltd., Nobleton, Ontario, Canada). We have used event related, short duration and low-intensity boxcar GVS to avoid the lateralized cueing due to the strong sensation under the cathode during stimulation. Although we did not formally investigate the feelings induced by our stimulation, we know it was easily tolerable and not unpleasant for the participants. Some reported a slight feeling of rotation or ‘dizziness’. Importantly, 1 mA GVS is sufficient to provide effective vestibular stimulation, because it induces postural reflexes (Fitzpatrick and Day 2004). Carbon rubber electrodes (area 10 cm^2^) coated with electrode gel were placed binaurally over the mastoid processes and fixed in place with adhesive tape. The area of application was first cleaned and electrode gel was applied to reduce impedance. Both left anodal and right cathodal (Left GVS) and right anodal and left cathodal (Right GVS) configurations were used (Fig. 1B). Using this binaural bipolar configuration, GVS is known to increase the firing rate in vestibular afferents on the cathodal side and to decrease the firing rate on the anodal side (Goldberg et al. 1984). We also applied Sham stimulation using electrodes placed on the left and right side of the neck, about 5 cm below the GVS electrodes (Lopez et al. 2010; Ferrè et al. 2013), with a left anodal and right cathodal configuration (Fig. 1B). Although the electrodes placed on the neck might induce different skin sensations and stimulate different nerves than the one placed behind the ears, this Sham stimulation can evoke similar tingling skin sensations to GVS, and so functioned as a control for non-specific effects.

### Experimental procedure

Verbal and written instructions were given to participants at the beginning of the experiment. The experiment was administered in sitting position to reduce postural effects of GVS. The head was in a neutral posture, i.e., neither flexed nor tilted. Participants were asked to fixate the computer monitor with hands on a keyboard. Electrodes for GVS and Sham stimulation were placed at the beginning of the session and remained in place for the entire duration of the experiment. The electrodes and the polarity of stimulation were selected under computer control.

Stimuli for the Misalignment Paradigm were similar to our previous studies (Alsmith et al. 2017; Longo et al. 2020) and are shown in Fig. 1A. In each trial, the image of an avatar appeared. Stimuli were presented on a 24-inch monitor (1024 × 768 pixels) located approximately 40 cm in front of the participant under control of a custom LabVIEW (National Instruments Corporation, www.ni.com/labview; Bitter et al. 2006) script. On each block of trials, the position of the avatar’s torso was held constant with the torso (200 pixels in width, 10.63° visual angle) centred on the monitor, oriented toward one of five compass directions (E, NE, N, NW, W), with the head rotated 45° to either the right or left. This resulted in ten different orientations and each of the ten positions was presented once per vestibular stimulation condition. Presenting the body in different orientations ensured that participants were basing their judgments on a reference frame centred on the avatar depicted, rather than on their own body, visual field, or any other external cues. In each trial, a ball (21 pixels in diameter, 0.8°) appeared at nine angles evenly spaced between − 60° and + 60° degrees from the line midway between the head and torso. Participants were asked to make simple spatial judgments about the location of the ball with respect to the avatar depicted. For each angle, there were two distances of the ball from the person, *Near* (6.39° from the centre of the head), and *Far* (19.17°). This allowed to investigate a potential spatial gradient in the vestibular modulation of head and torso references. In the Misalignment Paradigm, the most informative judgments are the ones in which the ball appears at the three centre angles (0°, 15°, − 15°). In these trials, the ball could be judged to the avatar’s right or to the avatar’s left in function of the references used by the participant. Please consider Fig. 1A, ball b. If the torso is used as origin of the egocentric reference frame, the ball is to the avatar’s left; if the head is the origin, it is to their right. To maximize the number of most informative judgments, within each distance the three centre angles (0°, 15°, − 15°) were each presented three times, the next three most extreme on each side (30°, 45°, 60°, − 30°, − 45°, − 60°) were each presented twice, and the most extreme angles (75°, 90°, − 75°, -90) were each presented once for each vestibular stimulation condition (Left-GVS, Right-GVS and Sham stimulation). A total of 75 trials was presented. Left GVS, Right GVS or Sham stimulation was applied for 3 s in each trial. The stimulation was delivered after 2 s from the beginning of the trial, then after 1 s the ball appeared for 2 s. After that, the ball disappeared and GVS was also turned off. Participants were instructed to “judge whether the ball is to the person’s left or to their right”. They made responses by pressing the ‘q’ key on the keyboard with the left index finger if they judged the ball as being to the person’s left and the ‘p’ key with their right index finger if they judged it as being to the person’s right. Participants were instructed to make the judgments within the 2 s time window in which the stimulation was on and the ball displayed on the screen. To avoid aftereffects of GVS on the subsequent trial, there was an inter-trial-interval of 3 s. The body remained on the screen during the interval. GVS conditions were applied in separate blocks and the order of GVS conditions was counterbalanced. The experiment lasted about 1 hour.

### Analysis

The analysis was similar to that used in our previous study (Alsmith et al. 2017; Longo et al. 2020). Best-fitting cumulative Gaussian functions were fit using maximum-likelihood estimation for each participant in each condition using the Palamedes toolbox for MATLAB (Mathworks, Natick, MA, Prins and Kingdom (2009), available online at: http://www.palamedestoolbox.org). To isolate contributions of the head, we analysed responses as a function of angular deviation of the ball from an axis aligned with the torso, comparing the conditions in which the head was rotated to the right vs. to the left. Conversely, to isolate contributions of the torso, we analysed responses as a function of angular deviation of the ball from an axis aligned with the head, comparing the conditions in which the torso was rotated to the right vs. to the left. For each psychometric function, the Point of Subjective Equality (PSE) was estimated. We quantified the contribution of the head and of the torso by calculating the *PSE Shift* for each body part, defined as the difference in PSE between the conditions in which the relevant part was rotated to the left and to the right. Because the total PSE Shift for the head and for the torso must add to 90°, we also calculated the proportionate weighting given to each of the two body parts by dividing the PSE Shift by 90°.

## Results

**Fig. 2:**
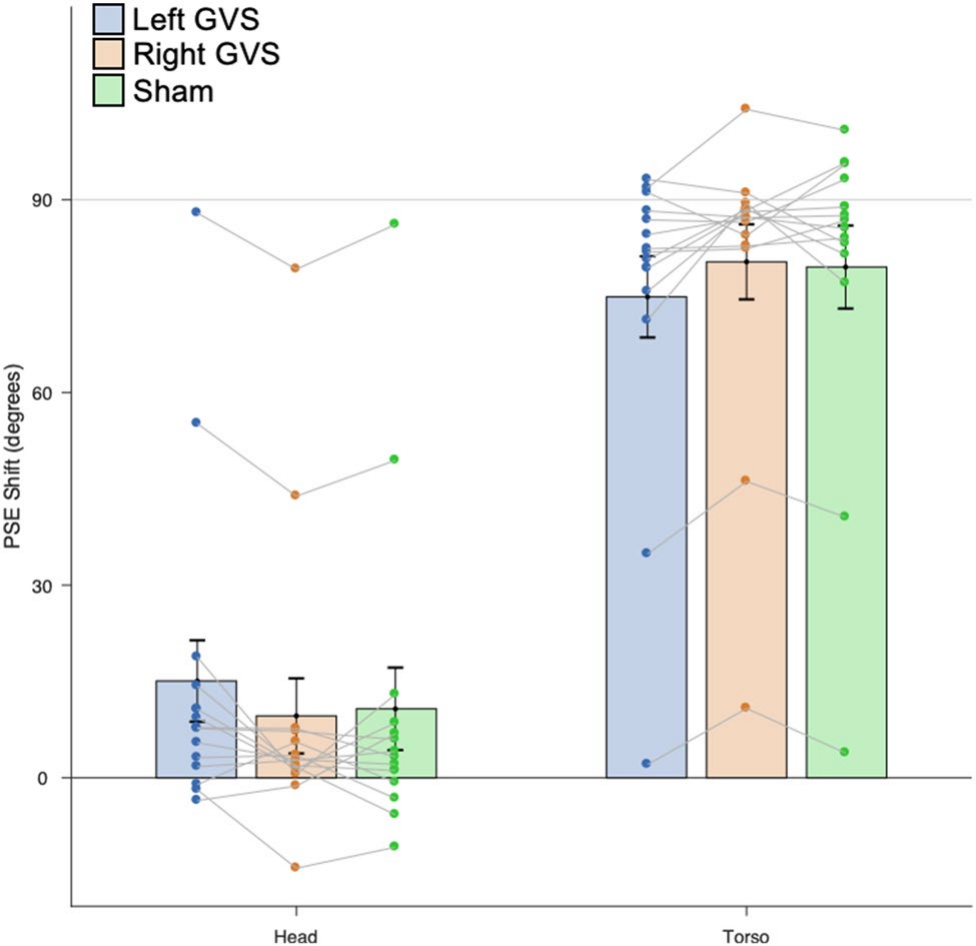
PSE shift as function of vestibular stimulation conditions. The contribution of the head and of the torso was quantified by calculating the *PSE Shift* for each body part, defined as the difference in PSE between the conditions in which the relevant part was rotated to the left and to the right. Because the total PSE Shift for the head and for the torso must add to 90°, we also calculated the proportionate weighting given to each of the two body parts by dividing the PSE Shift by 90°. PSE shifts in each experimental conditions are presented as single subject data and average across participants. Bars indicate standard error

Psychometric functions showed an excellent fit to the data, with a mean *R*^2^ of 0.960 (range 0.683–1). As no effects of distance were found, the near and far distances were collapsed in Fig. 1C and subsequent analyses. Individual subjects and Mean PSE Shift data as function of vestibular stimulation conditions (Left GVS, Right GVS, Sham) are reported in Fig. 2.

There were clear contributions of the torso in the Sham stimulation (Mean PSE Shift 79.5°, Mean weighting 0.884), *t*(14) = 12.30, *p* < 0.0001, *d*_*z*_ = 3.18, Left GVS (Mean PSE Shift 74.9°, Mean weighting 0.832), *t*(14) = 11.84, *p* < 0.0001, *d*_*z*_ = 3.06, and Right GVS (Mean PSE Shift 80.3°, Mean weighting 0.893), *t*(14) = 13.74, *p* < 0.0001, *d*_*z*_ = 3.55, conditions. Contributions of the head were less clear than in our previous study (Alsmith et al. 2017). While there was a significant contribution of the head in the Left GVS condition (Mean PSE Shift 15.1°, Mean weighting 0.168), *t*(14) = 2.38, *p* = 0.03, *d* = 0.61, this did not reach significance in the Sham Stimulation (Mean PSE Shift: 10.7°, Mean weighting: 0.119), *t*(14) = 1.67, *p* = 0.12, *d* = 0.43, or Right GVS (Mean PSE Shift 9.6°, Mean weighting 0.107), *t*(14) = 1.65, *p* = 0.12, *d* = 0.43, conditions.

An ANOVA on PSE Shifts revealed a main effect of Body Part (Head, Torso), *F*(1, 14) = 29.35, MSE = 3382.90, *p* < 0.0001, η_p_^2^ = 0.68. Critically, this effect was modulated by a significant interaction between body part and stimulation condition, *F*(2, 28) = 5.78, MSE = 43.84, *p* = 0.008, η_p_^2^ = 0.29. There were no other significant main effects or interaction (p > 0.05). Follow-up testing using the Bonferroni Holm procedure showed that Left GVS increased the weight given to the head compared to both Right GVS, *t*(14) = 3.03, *p* = 0.009, *d*_*z*_ = 0.78, and Sham stimulation, *t*(14) = 2.48, *p* = 0.02, *d*_*z*_ = 0.64. There was no differences between Right GVS and Sham stimulation *t*(14) = 0.71, *p* = 0.49.

## Discussion

The vestibular system provides a head-related signal, constantly detecting head acceleration and orientation in the three-dimensional space (Lackner and DiZio 2005). Our data showed that the weight given to the head in determining an object’s location was increased during left anodal and right cathodal GVS, compared to the opposite GVS polarity and Sham stimulation. That is, the polarity of GVS, which preferentially activates vestibular areas in distinct cerebral hemispheres, had differential effects on the weighting of head and torso in egocentric spatial judgements.

Previous research investigating the contribution of different body parts to egocentric spatial judgements showed that both the head and the torso contribute to the determination of egocentric representation, with slightly greater reliance on the torso (Alsmith et al. 2017). In our knowledge, no studies focused on whether the contribution of these body parts to egocentric spatial judgments is a stored representation reflecting semantic knowledge about the body morphology, or a dynamic, online updated sensory representation, perhaps reflecting the saliency of afferent sensory signals. Our results showed a change in the weight given to the head and torso when artificial vestibular stimulation was delivered to enhance vestibular processing. This suggests that the saliency of afferent sensory signals plays a role in the weight given to different body parts while making egocentric spatial judgements.

GVS polarity-dependent differences in postural, sensorimotor and cognitive functions have been demonstrated both in healthy volunteers and in brain damaged patients. This might arise if one polarity of GVS has stronger effects in the brain, perhaps reflecting a cerebral dominance for vestibular processing. Accordingly, neuroimaging studies have identified an asymmetry in the cortical vestibular system, suggesting that the cortical vestibular network is primarily located in the non-dominant right hemisphere in right handed participants (Dieterich et al. 2003; Bense et al. 2001; Suzuki et al. 2001; Janzen et al. 2008). Therefore, the polarity-specific influence of left anodal and right cathodal GVS on the head weight in the Misalignment Paradigm may be related to modulations of mechanisms encoding egocentric representations in the right hemisphere. However, the mechanism that links GVS polarity effects to cortical dominance remains still unclear. In particular, one might imagine that the dominant right hemisphere vestibular projections could be activated by both left anodal and right cathodal GVS and right anodal and left cathodal GVS (Eickhoff et al. 2006a, b), yet we found effects only of left anodal and right cathodal GVS. However, fMRI studies identified a relatively strong activation of the right hemisphere during left anodal and right cathodal GVS compared to the opposite polarity (Fink et al. 2003). Thus, right anodal and left cathodal GVS may have simply been not strong enough to modulate the egocentric frame of reference.

Clinical reports have shown that a unilateral lesion to the vestibular peripheral organ yields to a tonic imbalance in vestibular processing which may contribute to postural, balance and gait problems, including head and trunk tilt deviation to the lesioned side (Borel et al. 2008). Alterations in  the representation of body orientations have also been described after unilateral vestibular loss (Saj et al. 2013). Interestingly, only patients with left vestibular loss, a vestibular asymmetry which is consistent to that created by left anodal and right cathodal GVS, showed more severe biases in the representation of self-orientation in space. Taken together these results converge with a right hemispheric dominance for space representation, as well as a right hemispheric dominance of cortical vestibular projections (Bottini et al. 1994; Dieterich et al. 2003).

Previous studies have investigated the relation between body parts orientation and spatial attention. Grubb and Reed (2002) observed a pseudoneglect bias in a covert attention task by leftward rotation of the torso. Hasselbach-Heitzeg and Reuter-Lorenz (2002) also found that rightward rotation reduced response times for targets on the right. Potentially, GVS could have affected egocentric spatial judgements indirectly through attentional or arousal mechanisms, rather than through any direct effects on egocentric representation. Left anodal and right cathodal GVS produces shift in spatial attention toward the left space, whereas right anodal and left cathodal GVS induces an attentional bias toward the right space (Ferrè et al. 2013). In Left GVS trials, the attention of participants might have been shifted toward the left hemispace. However, this general shift could not explain the clear interaction we found between GVS polarity and head vs torso judgements. This interaction was due to the head references only: there was no difference between Left GVS and Right GVS in the torso judgments. Thus, an explanation based on shifts of spatial attention cannot readily account for our results. Accordingly, Rorden et al. (2001) also found that inducing illusions of torso rotation did not produce effects in attentional orientation.

The Misalignment Paradigm suggests that egocentric spatial judgments involve the use of reference frames centred both on the head and torso, with differences between people in the use of these body parts as anchor (Alsmith et al. 2017; Longo et al. 2020). Although the factors that may drive individual differences in the use of these body parts or in the use of a weighted combination of head and torso are not yet entirely clear, we have recently shown an high stability across time (Longo et al. 2020). Participants re-tested on the Misalignment Paradigm several months after the original test showed a strong correlation between the two testing sessions in their use of head vs. torso references. However, further research might focus specifically on the drivers of these differences.

It is important to note that the Misalignment Paradigm measured egocentric spatial judgments using a third-person perspective taking task in which participants are explicitly being asked to make judgments of spatial position with respect to a seen avatar. Thus, the effects of vestibular stimulation on head and torso references do not involve location of targets directly from the participant's first-person perspective. Recent studies have demonstrated vestibular modulation of third-person perspective taking both in healthy participants (Deroualle et al. 2015) and vestibular patients (Deroualle et al. 2019). Interestingly, only patients with left vestibular loss presented altered third-person perspective taking compared to controls. No impairment was observed in first-person perspective taking and the 3D objects mental imagery abilities (Deroualle et al. 2019). These results are in agreement with the findings of the present study, supporting the importance of a right cortical vestibular network for the cognitive representation of the body in space.

In conclusion, our study highlights vestibular contributions to egocentric representation. Egocentric spatial judgments rely on a weighted combination of reference frames centred on at least two different parts of the body: the head and torso. We have shown that afferent vestibular signals modulates the relative weightings of head and torso, showing that vestibular information contributes to computation of egocentric representations.

## Data Availability

The datasets generated during and/or analysed during the current study are available from the corresponding author on reasonable request.
